# There is Only One Valid Definition of Clearance: Critical Examination of Clearance Concepts Reveals the Potential for Errors in Clinical Drug Dosing Decisions

**DOI:** 10.1208/s12248-021-00591-z

**Published:** 2021-05-10

**Authors:** Leslie Z. Benet, Jasleen K. Sodhi, George Makrygiorgos, Ali Mesbah

**Affiliations:** 1 Department of Bioengineering and Therapeutic Sciences, Schools of Pharmacy and Medicine, University of California, San Francisco, California 94143-0912 USA; 2 Department of Chemical and Biomolecular Engineering, University of California, Berkeley, California 94720-1462 USA

**Keywords:** clearance, chemical reaction engineering models, well-stirred model

## Abstract

Drug dosing decisions in clinical medicine and in introducing a drug to market for the past 60 years are based on the pharmacokinetic/clinical pharmacology concept of clearance. We used chemical reaction engineering models to demonstrate the limitations of presently employed clearance measurements based upon systemic blood concentration in reflecting organ clearance. The belief for the last 49 years that *in vivo* clearance is independent of the mechanistic model for organ clearance is incorrect. There is only one valid definition of clearance. Defining organ clearance solely on the basis of systemic blood concentrations can lead to drug dosing errors when drug effect sites reside either in an eliminating organ exhibiting incremental clearance or in a non-eliminating organ where intraorgan concentration is governed by transporter actions. Attempts to predict clearance are presently hampered by the lack of recognition that what we are trying to predict is a well-stirred model clearance.

**Supplementary Information:**

The online version contains supplementary material available at 10.1208/s12248-021-00591-z.

## INTRODUCTION

Clinical medicine drug dosing decisions are traditionally based on pharmacokinetic clearance concepts. In addition, the recommended dose and dosing interval for a new molecular entity (NME) introduced to the market by a pharmaceutical sponsor relies on pharmacokinetic drug clearance. Drug dosing adjustments in disease states (e.g., renal failure and hepatic dysfunction), drug dosing changes required due to drug–drug interactions in a patient, precision medicine dosing adjustments due to pharmacogenomic variance, and physiologic differences between individuals are all governed by the clearance and changing the clearance of a drug. Measured systemic drug concentrations (*C*_*systemic*_) are most often used in making these drug dosing decisions because of its direct relationship with clearance (*CL*) as given by

1 $$ \mathrm{Amount}\ \mathrm{eliminated}\ \mathrm{per}\ \mathrm{unit}\ \mathrm{time}= CL\cdot {C}_{systemic} $$

where the amount eliminated per unit time is measured in units of mass/time, *C*_*systemic*_ in units of mass/volume, and thus the units of *CL* are volume/time. Equation 1 embodies the definition of clearance, i.e., the amount eliminated divided by the exposure (systemic concentration) driving that elimination. Clearance is a measure of the volume of systemic fluid (e.g., blood, plasma) flowing through the body’s organs of elimination that is completely removed of the drug per unit time. Here, we will deal only with linear systems (i.e., no saturable processes) for simplicity. Note that *in vivo* in humans, clearance cannot be calculated using Eq. 1, since it is not possible to measure the amount eliminated per unit time where elimination is via metabolism or biliary excretion, except at steady state. However, for renal clearance where the amount eliminated per unit time in the urine is measurable, clearance can be calculated by Eq. 1. Clearance for all drugs can be determined at steady state where rate in equals rate out; thus, Eq. 1 is modified in that dosing rate (rate in, units of mass/time) equals *CL* (units of volume/time) multiplied by steady-state concentration (*C*_*ss*_, units of mass/volume) (rate out). More usually, apparent body clearance is calculated following a single dose of the drug (units of mass), where the fraction available (*F*) of the dose reaching the systemic fluids (amount eliminated) is divided by the drug exposure in the systemic fluids (the exposure driving that elimination), measured as the area under the curve of the systemic drug concentrations (AUC, units of mass·time/volume) over all time is obtained by integrating Eq. 1 from time zero to infinity yielding Eq. 2.

2 $$ CL=\frac{F\cdot \mathrm{Dose}}{\mathrm{AUC}} $$

The drug bioavailability (*F*) is assumed to be unity when drug is given as an intravenous dose directly into systemic circulation but can be and is often less than unity when alternate routes of administration are employed. For example, following oral dosing *F* can be influenced by the extent of drug absorption from the gastrointestinal tract and by the first-pass metabolism in the intestinal membranes and the liver, because when drug is administered orally, it must first pass through the gastrointestinal membranes and the liver before reaching the heart and becoming measurable in the systemic fluids. All published clearance values are either determined by the steady-state variance of Eq. 1 described earlier or by Eq. 2 based on integrated measurements of systemic drug concentrations following a single dose.

In 1972, Rowland (1) introduced what appeared to be a very useful clearance relationship for an organ of elimination. Let us consider here the liver as an isolated organ at steady state, where it is only possible to measure the systemic concentration entering the liver (*C*_*in*_), the systemic concentration exiting the liver (*C*_*out*_), and the hepatic blood flow (*Q*_*H*_). The relationship is not limited to drugs that are only eliminated by the liver but is operational for any organ where elimination is rate limited by blood flow but would not be relevant for drugs that are degraded in the blood by hydrolysis, esterases, and proteases (e.g., nitroglycerin, aspirin, penicillamine) and thus are not rate limited by organ blood flow. The hepatic clearance (*CL*_*H*_) as proposed by Rowland (1) is

3 $$ {CL}_H={Q}_H\cdot \frac{C_{in}-{C}_{out}}{C_{in}}={Q}_H\cdot ER $$

where *ER* is the extraction ratio, the fraction of drug relative to *C*_*in*_ that is cleared/eliminated upon each passage through the liver. But how is the Eq. 3 liver clearance related to the definition of clearance given in Eqs. 1 and 2 or, alternatively, is there more than one definition of clearance? The purpose of this paper is to demonstrate based on chemical reaction engineering principles how Eq. 3 is or is not consistent with the Eqs. 1 and 2 definitions of clearance, an important aspect of the widely used Eq. 3 that has been ignored. There is only one mechanistically meaningful definition of clearance, but this has not been recognized in pharmacokinetics leading to misuse and misinterpretation of clearance concepts. Note that Eq. 3 appears to be a restatement of Eq. 1 where clearance is calculated as the amount eliminated per unit time at steady state, i.e., *Q*_*H*_ ∙ (*C*_*in*_ − *C*_*out*_), divided by a specific systemic concentration, *C*_*in*_. There are many potential models of organ elimination (that are based on reaction engineering models); however, Eq. 3 has been generally assumed to be model independent (2).

Drug development is an extremely expensive and time-consuming process with an unacceptable and very poor success rate. Since clearance is such a critical parameter, it seems obvious that if we could predict an NME’s clearance prior to dosing the drug to humans or even animals, this could markedly speed the drug development process (3, 4). However, even when conducting what should be relevant and translatable *in vitro* measures of drug elimination with human hepatocytes and microsomes, successful prediction of metabolic clearance within 2-fold of measured values fails 60–80% of the time based on numerous evaluations of IVIVE success (5–7)*.*

In the present manuscript, we show that Eqs. 1–3 have only been stated as facts, while the derivations and inherent assumptions of these relationships have not been examined. Clearance concepts in pharmacokinetics are thought to be analogous to chemical reaction engineering representations of reactors for which only entering and exiting reactant amounts are known. Using the basic models and principles of reaction engineering, we have investigated the various pharmacokinetic models of organ clearance and show that many universally accepted principles of pharmacokinetics concerning clearance are not correct. We demonstrate that Eqs. 1–3 inherently include assumptions that prevent the successful prediction of clearance by presently universally employed methodologies.

## THE CHEMICAL REACTION ENGINEERING MODELS THAT ARE BELIEVED TO APPLY TO PHARMACOKINETIC CLEARANCE DETERMINATIONS

In the field of pharmacokinetics, representation of the major elimination organ, the liver, has been approached very similarly to how the field of reaction engineering has modeled reactors to understand the rate of chemical reactions within the reactor. It is believed that chemical reactor models directly translate to the pharmacokinetic models of organ elimination. Various models based on conservation laws and flow patterns have been developed to characterize what happens within a chemical reactor (8).

**Fig. 1:**
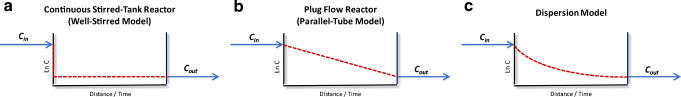
Chemical engineering reaction (pharmacokinetic) models at steady state (logarithmic concentration on Y-axis)

The simplest model that is utilized, designated the continuous stirred-tank reactor (CSTR), assumes that the reaction is homogenous (8), i.e., the reactants are uniformly distributed within the reactor (perfect mixing). That is, there is no spatial distribution of concentration within the reactor. The CSTR model is depicted in Fig. 1a and exhibits infinite dispersion or perfect mixing within the reactor (i.e., the same concentration of substrate is present at all points within the reactor). The Y-axis of the figure is given in logarithmic concentrations, while the X-axis is the reaction length, but at steady state when the rate of accumulation of substrate is constant (i.e., steady state is reached), the perfect mixing makes the use of either length or time possible in the X-axis. In other words, distance and time are not independent, and thus, the X-axis may be either length or time in Fig. 1a–c. The CSTR is one extreme case of a chemical reactor model. The other extreme case is the plug flow reactor (PFR) model where the concentration of substrate within the reactor decreases by a first-order process throughout the reactor (8) as depicted in Fig. 1b. The PFR model exhibits zero dispersion within the reactor (i.e., no mixing) such that the reaction occurs throughout the reactor and concentrations change incrementally. That is, concentrations decrease throughout the reactor, resulting in a concentration gradient.

There is an infinite number of dispersion models between the two extreme cases of CSTR and PFR that follow linear kinetics and are characterized by different dispersion numbers (see Supplementary material) ranging from zero to infinity, one of which is depicted in Fig. 1c. In the Supplementary material, we show that Eq. 4 describes the mass–balance relationship for both of the extreme cases, CSTR and PFR:

4 $$ \frac{\mathrm{Total}\ \mathrm{rate}\ \mathrm{of}\ \mathrm{reaction}}{\mathrm{Rate}\ \mathrm{of}\ \mathrm{delivery}\ \mathrm{to}\ \mathrm{the}\ \mathrm{reactor}}=\frac{\left({C}_{in}-{C}_{out}\right)}{C_{in}} $$

This may at first seem contradictory since it can be seen in Fig. 1 that the concentration of substrate in the reactors differs from model to model. But in terms of the total rate of reaction divided by the rate of delivery to the reactor, Eq. 4 holds for the extreme cases, CSTR and PFR models (derived in Supplementary material), and should also be true for all models with varying degrees of dispersion in between, although this derivation has not been explicitly presented.

## INAPPROPRIATE TRANSLATION OF THE CHEMICAL REACTION ENGINEERING MODELS TO PHARMACOKINETIC MODELS OF ORGAN ELIMINATION AND THE CORRECT CLEARANCE RELATIONSHIPS

The discrepancy between chemical reaction engineering and pharmacokinetics is that the mass–balance relationship presented in Eq. 4 that holds for all reactor models has been misconstrued when applied to pharmacokinetic clearance relationships. Equation 4 does not give any information on how drug concentration changes between entering and exiting concentrations within an organ; it merely indicates that no matter how one chooses to model the relationship between entering and exiting concentrations, mass balance is conserved, a condition that is valid for both fields. However, in pharmacokinetics, the substrate concentrations within an organ of elimination can have a critical clinical significance when such concentrations drive pharmacological outcomes. Therefore, the differing assumptions of how concentrations are modeled between each model can have significant implications on predictions of drug pharmacologic outcomes. Although clearance is not measured in chemical reactor modeling, different models are characterized in terms of mean residence time, the average time that a substrate molecule will remain unreacted within the reactor. It is well documented in chemical engineering (8) that the mean residence time will be different for the different models depicted in Fig. 1. As with clearance in pharmacokinetics, there is only one definition of mean residence time, the volume of distribution of the drug in the system being analyzed divided by the clearance of the drug from that system. As in chemical reaction engineering where the volume of the reactor is independent of the model, the volume of distribution in the different models of hepatic elimination is assumed to be the same. Therefore, the different mechanistic liver clearances for the equivalent pharmacokinetic models of organ elimination as designated in Fig. 1**(**well stirred, parallel tube, and dispersion) will result in different mean residence times for each of these models.

Of, course, the right-hand side of Eq. 4 is the extraction ratio (*ER*) of Eq. 3. For almost 50 years, the field of pharmacokinetics has incorrectly believed that this relationship indicates that *CL* in Eq. 3 is model independent, when in fact Eq. 4 simply indicates mass–balance for all of the reactor models. But medical practitioners, clinical pharmacologists, and pharmacokineticists are not making drug dosing decisions based on the mass–balance of the total rate of reaction in the body relative to the rate of drug delivery. Rather clinical dosing decisions are always based on the drug exposure that the patient experiences, that is, the concentrations measured and the integral of those concentrations over time since the pharmacodynamic effect will be related to drug exposure (not the relative rate of drug removal). Similarly, for the three models of organ elimination in Fig. 1, it is obvious that the resulting drug exposure in the liver is very different, just as the mean residence time for the three models is very different. Therefore, the drug clearance, which is a measure of the amount of drug eliminated divided by the exposure driving that elimination (Eq. 2), would be very different.

Rowland *et al*. (9) proposed that Eq. 5 defines the clearance of the well-stirred model (WSM) of Fig. 1a.

5 $$ {CL}_{H,\mathrm{WSM}}={Q}_H\cdot \frac{\left({C}_{in}-{C}_{out}\right)}{C_{in}} $$

Note in Eq. 5 that the concentration driving elimination is not the concentration within the liver, as can be seen in Fig. 1a, but rather the concentration entering the liver. We show in the Supplementary material derivation why Eq. 5 is consistent with the well-stirred model. Figure 1b in terms of clearance concepts will translate into Eq. 6, where the amount of drug eliminated (which is model independent) will be divided by the average concentration at steady state driving the elimination. For first-order elimination in the liver, the parallel tube model (PTM) of pharmacokinetics, the average concentration at steady state will be $\frac{\Big({C}_{in}-{C}_{out\Big)}}{\ln \frac{C_{in}}{C_{out}}}$. Thus, hepatic clearance (derived in Supplementary material) will be given by

6 $$ {CL}_{H,\mathrm{PTM}}={Q}_H\cdot \mathit{\ln}\frac{C_{in}}{C_{out}} $$

For Fig. 1c, representing all dispersion models (DM), hepatic clearance will be given by Eq. 7, where the average concentration at steady state will be dependent on the dispersion constant.

7 $$ {CL}_{H,\mathrm{DM}}={Q}_H\cdot \frac{\left({C}_{in}-{C}_{out}\right)}{C_{\mathrm{average}}} $$

Thus, in pharmacokinetics, all of the models of organ elimination will yield different mechanistic liver clearance values (Eqs. 5–7) due to the differences in drug concentrations within the organ (Fig. 1), although the ratio of the total rate of reaction to the rate of drug input (Eq. 4) would be the same for the extreme cases of WSM and PTM and intermediate dispersion models. Of these three mechanistic clearance relationships, the well-stirred model (Eq. 5) is identical to Eq. 3, that is, equivalent to the equation presented without derivation by Rowland (1) in 1972, where clearance is equal to organ blood flow multiplied by the extraction ratio. At that time, it was incorrectly believed that the equation was model independent, and this belief has persisted throughout the field of clinical sciences for half a century.

## WHY WOULD PHARMACOKINETICISTS/CLINICAL PHARMACOLOGISTS BELIEVE THAT EQ. 3 WAS MODEL INDEPENDENT? MOREOVER, WHY IS THAT CONTENTION UNTRUE?

As detailed earlier, the inappropriate understanding of how to translate chemical reaction engineering concepts led to the belief that, because the ratio (total rate of reaction/rate of delivery to the reactor) is equal for each of the various reactor models that it was valid to assume this also held true for the ratio of organ clearance/organ blood flow. Further, unfounded assertions by leading scientists in the field persisted in this belief that Eq. 3 was model independent. Rowland and Pang wrote (10) that *C*_*in*_ in the denominator of Eq. 3 was only a “reference concentration” implying no specific model and that Eq. 3 was model independent, consistent with their statement in 1977 that by definition, Eq. 3, without any derivation, defines clearance (2). This contention of Rowland and Pang of a “reference concentration” had never been previously stated and was only invoked (10) when we pointed out that, by analogy to chemical reaction engineering, Eq. 3 was only consistent with the well-stirred model (11). The “reference concentration” of Rowland and Pang (10), where *C*_*in*_ is not driving elimination, implies that there is a second definition of clearance that has been invoked for an isolated perfused organ.

If Eq. 3 was, in fact, model independent, there should be experimental data in the literature supporting alternate models of hepatic elimination. The only way that the model dependence can be experimentally tested *in vivo* is to carry out isolated perfused rat liver (IPRL) studies where blood flow and protein binding can be varied while measuring *C*_*in*_ and *C*_*out*_. There are many, many more papers in the literature discussing the theoretical basis for the various models of hepatic elimination, as compared to papers actually providing experimental studies testing those theories. Moreover, there was no paper in the literature critically reviewing those experimental studies. In our recent paper (12) “Are There Any Experimental Perfusion Data that Preferentially Support the Dispersion and Parallel Tube Models Over the Well-Stirred Model of Organ Elimination?” we conclude “Thus, in response to the title of this manuscript, we find no experimental data that reasonably or unambiguously support preference for the dispersion or parallel-tube models versus the well-stirred model of organ elimination when only entering and exiting drug concentrations are available. However, there are data that unambiguously show that *C*_*out*_/*C*_*in*_ measurements with changing blood flow and protein binding can only be fit by the well-stirred model.”

Where are the flaws in the statement that *C*_*in*_ in the denominator of Eq. 3 is only a “reference concentration” (10), making Eq. 3 model independent, as had been considered for 49 years? First, “reference concentration” is a new concept. The concept has never been previously proposed in pharmacokinetics. There is no theoretical basis for a reference concentration defining clearance. Second, all pharmacokinetic relationships must be derivable, and the apparent body clearance as used clinically has always been defined as the amount eliminated divided by the concentration driving that elimination as given in Eq. 2 and the steady-state equivalent of Eq. 1. The “reference concentration” approach implies there is a noncongruent second definition of clearance. Third, all IPRL experimental data measuring *C*_*in*_ and *C*_*out*_ appear to only fit the well-stirred model (12)*.*

## ALL CLEARANCE MEASURES USED IN CLINICAL MEDICINE ARE ONLY CONSISTENT WITH EQS. 1 AND 2

But there remains an even greater universally unrecognized misinterpretation in the field of pharmacokinetics with respect to clearance via organs of elimination. No matter what representation of drug elimination is proposed, a physiologic relevant liver model, such as the dispersion model, or an unphysiologic “well-stirred” model, either for IPRL studies or for whole-body measures of elimination, the outcome appears consistent with Eq. 3, designated by Rowland *et al*. (9) as the well-stirred model. Furthermore, Eq. 3 appears to work well clinically, apparently for all drugs, setting an upper limit of organ blood flow. In an attempt to differentiate the components of clearance, Eq. 8 was derived (9, 13) in the 1970s (derived in a more direct fashion in Supplementary material) to predict the outcome of changes in organ blood flow (*Q*_*H*_), fraction unbound to proteins in the blood (*f*_*u,B*_), and the inherent activity of the metabolic enzymes to eliminate/metabolize the unbound drug, designated as intrinsic clearance (*CL*_*int*_):

8 $$ {CL}_{\mathrm{organ},\mathrm{WSM}}={Q}_{\mathrm{organ}}\cdot \frac{f_{u,B}\cdot {CL}_{int}}{Q_{\mathrm{organ}}+{f}_{u,B}\cdot {CL}_{int}} $$

As we noted earlier, all clearance measurements utilized in clinical medicine, in recommending the dose and dosing interval for an NME brought to the market by a pharmaceutical sponsor, in making drug dosing adjustments in disease states (e.g., renal failure and hepatic dysfunction), in drug–drug interactions in a patient, and attempting to make precision medicine dosing adjustments due to pharmacogenomic variance and physiologic differences, are all determined utilizing the basic relationships in Eqs. 1 and 2. As we also noted, all pharmacokinetic relationships can be derived, and no one has ever evaluated the derivation of Eqs. 1 and 2 and the inherent assumptions of these equations. We do so here for the first time. Clearance, as it is currently calculated in humans, is assumed to be only driven by observable systemic concentrations in Eqs. 1 and 2, and as a result, these defining equations assume that the unmeasurable concentrations within the organ have no influence on clearance. The implication is that Eqs. 1 and 2 assume that there is no incremental elimination in calculating clearance at steady state or when integrating concentrations over all time for a single dose.

Reviewing the history of clearance further elucidates why this observation has never been made previously. Concepts of clearance first evolved in renal physiology with the “intact nephron hypothesis” (14) of Bricker in 1960, proposing that the health of all kidney functions could be based on the passive glomerular filtration rate (GFR), where the clearance measure of creatinine, the endogenous product of muscle breakdown, was used to evaluate GFR (15)*.* The well-stirred model approach works well for renal elimination of drugs as passive glomerular filtration does not involve incremental processes within the kidney. It seemed very plausible to then carry over this relation to the liver and for liver clearance to be calculated by the total amount of drug eliminated divided by AUC, the systemic exposure. But by definition, linear metabolism is incremental (e.g., a first-order process more consistent with the parallel tube model of hepatic elimination), and physiologically, biliary excretion is a function of the concentration within the liver. The discrepancy for liver elimination (including both metabolism and biliary elimination) is that calculations of hepatic clearance are always based only on measured systemic concentrations. Based on the derivations here, such an approach inherently assumes that only systemic concentrations are the driving force for hepatic elimination. As such, incremental metabolism and biliary elimination are not considered.

## THE UNIVERSALLY UNRECOGNIZED ASSUMPTION IN CLINICAL MEDICINE, CLINICAL PHARMACOLOGY, AND PHARMACOKINETICS

We emphasize that all pharmacokinetic-based drug dosing decisions and adjustments of drug dosing in disease states, drug–drug interactions, and pharmacogenomic and physiologic variance are based on systemic drug concentration measurements. There is no consideration of potential processes that occur within organs of elimination. Therefore, these drug dosing decisions are only consistent with clearance calculated where the concentrations driving elimination are those entering the organ of elimination, the measured systemic concentrations in Eqs. 1 and 2. This means that presently employed drug dosing decisions will only be valid when pharmacodynamic response (such as therapeutic efficacy or toxicity) is proportionate to systemic concentrations as seen for a dose change or when there is a constant relationship between systemic concentrations and intraorgan concentrations such as differences due to partitioning, protein binding, and differential pH. Under these conditions, the intraorgan concentration is proportionately related to systemic concentrations by a constant, but when the intraorgan concentrations change as the drug passes through the organ of elimination as in the parallel tube and dispersion models, these concentrations are not proportionately related to the measured systemic concentration by a constant. It is well recognized that drug–drug interactions affecting hepatic uptake transporters can markedly change systemic drug concentrations and apparent body total clearance measurements for statins (HMG-CoA uptake inhibitors used to treat hyperlipoproteinemias in cardiovascular disease). However, these drug–drug interactions and systemic clearance changes will have little or no effect on liver intracellular drug concentrations and cardiovascular therapeutic outcome (16), but they can be relevant for predicting muscular toxicities of the statins (myalgia and rhabdomyolysis) that appear to be related to systemic concentrations (17)*.* Thus, clinical dose adjustments of statins based on higher observed systemic concentrations may only serve to mitigate adverse events related to systemic concentrations; it, however, could lead to a lack of clinical efficacy due to reducing therapeutic intrahepatic concentrations, as noted by Varma *et al*. (18). Simulations to this effect have recently been conducted by our laboratory, indicating the expected changes in systemic and intrahepatic drug concentrations based on alterations in (a) hepatic uptake transporters, (b) basolateral efflux, and (c) hepatic metabolic and biliary intrinsic clearance, and further demonstrate this concept (19). Even more recently, it has been recognized that greater than 5-fold changes in plasma exposure of firsocostat, a therapeutic agent that reduces hepatic de novo lipogenesis (with drug target within the liver), as a result of a rifampin drug–drug interaction, did not alter its pharmacodynamic effect or intrahepatic exposure (20). Thus, the use of Eqs. 1–3 and 8 can lead to inappropriate drug dosing decisions for any therapeutic modality where intraorgan concentrations drive the therapeutic outcome, and these intraorgan concentrations are different than systemic concentrations due to incremental clearance or transporter effects.

Note, this differentiation between systemic and intraorgan concentrations is not limited only to organs of elimination as was demonstrated in the aforementioned paragraph. Systemic concentration changes will not predict intraorgan concentrations, and pharmacodynamic effects related to these intraorgan concentrations and for any organ that may be affected by transporters such as the brain, testes, thyroid gland, and placenta. The clinical impact of the inability of systemic clearance to predict intraorgan pharmacodynamic outcomes is not limited to the statins, and this discrepancy can also be observed for the antiepileptic drug lamotrigine and the antibiotic ceftriaxone. Neither the efficacy nor central toxicities of lamotrigine can be correlated with plasma concentrations (21), and therefore, commonly employed therapeutic drug monitoring efforts that inform drug dosing adjustments based on lamotrigine plasma concentration or apparent body clearance measurements will not be useful. Lamotrigine is one of the few metabolized antiepileptic drugs that is highly permeable and poorly soluble (class 2 Biopharmaceutics Drug Disposition Classification System drugs), where transporter effects on brain concentrations are believed to be clinically relevant (22). Lamotrigine is a substrate of two brain efflux transporters, breast cancer resistance protein (BCRP), and P-glycoprotein (P-gp), which would be expected to lead to marked differences in the brain *versus* plasma concentrations (23).

Ceftriaxone is a parenterally administered antibiotic that is used for a range of infections, including biliary tract infections. Biliary excretion and biliary clearance of ceftriaxone are not correlated with plasma concentrations of the drug over time (24, 25), so again, total systemic apparent clearance cannot be used to adjust dosing of this drug for its efficacy related to biliary tract infections or to mitigate the primary toxicological outcome related to ceftriaxone and formation of stones in the biliary tract. However, renal clearance of ceftriaxone is very highly correlated with plasma clearance (25), as explained earlier since GFR is highly related to creatinine plasma concentrations, and thus, plasma concentrations are expected to correlate well in predicting the formation of ceftriaxone-related kidney toxicities. We do recognize that the PK/PD relationship will follow the *E*_*max*_ model and that drug dosing changes are not always adjusted based on AUC changes alone. The principles we are addressing here are the simplified case at concentrations essentially below *EC*_50_.

A second very relevant outcome of the finding reported here is that almost all of the research attempting to predict *in vivo* clearance does not recognize the assumptions of Eqs. 1–3 and therefore today may hinder attempts to improve predictions of *in vivo* clearance. This includes IVIVE (*in vitro–in vivo* extrapolation to predict clearance), measurements of intracellular concentrations, utilization of alternate more physiologic models of hepatic elimination, and PBPK (physiologically based pharmacokinetic) models used extensively in predicting drug pharmacokinetics, where regulatory agencies such as the FDA have promulgated guidances (26, 27) of how the PBPK modeling and simulation analyses should be submitted for requests to waive the conduct of clinical studies. *In vivo* apparent clearance determinations are based only on systemic concentrations.

## THE IMPLICATIONS

- All *in vivo* measured drug pharmacokinetic clearance values are based only on measured systemic concentrations, not intraorgan concentrations.
- Thus, the Eq. 3 relationship, *CL* = *Q*_organ_ ∙ Extraction Ratio, is relevant and can be useful for all drugs where elimination is via blood flow limited organs where pharmacodynamic response is proportionately related to systemic concentrations.
- More physiologically realistic models of organ elimination provide no useful information in making drug dosing decisions in disease states, drug–drug interactions, or due to pharmacogenomic variance since clinical clearance determinations are only based on measurements of systemic drug concentrations.
- No models of hepatic elimination are relevant in predicting *CL*_*in vivo*_ in IVIVE approaches as intraorgan concentrations are not considered in *in vivo* clearance determinations.
- Measurements of intracellular concentrations (or their estimation if *K*_*p*,*uu*_ is known) will facilitate mechanistic understanding but often will not be useful for predicting apparent *CL*_*in vivo*_ when intraorgan concentrations decrease incrementally or are affected by transporters.
- The bottom-up approach in PBPK (i.e., utilizing *in vitro* measures to predict *in vivo* disposition), with the addition of fudge factors, may predict concentration-time curves, but the model-estimated clearance will not be the *CL*_*in vivo*_ due to the poor IVIVE predictability.
- Apparent clearance measurements based on systemic drug concentrations may not yield correct drug dosing decisions for those drugs where pharmacodynamic effects are a function of intraorgan drug concentrations and when intraorgan concentrations decrease incrementally (such as when elimination occurs in the same organ) or are affected by transporters.

## CONCLUSIONS

Pharmacokinetics and the use of clearance was a revolutionary advance in therapeutics allowing rational decisions about the appropriate dose and dosing interval, and how these doses should be adjusted as a function of disease states when additional drugs are dosed leading to clearance changes and dose adjustments necessary because of pharmacogenomic and physiologic variance. Clinically, it makes little difference that we did not recognize that these apparent clearance measurements were only consistent with systemic concentrations nor that we did not recognize that the similarity of chemical reactor models with respect to rates of reaction would not carry over to pharmacokinetic clearance models. But these drug dosing decisions may not be correct when the pharmacodynamic outcome is a function of intraorgan concentrations. Further, in our attempts to predict clearance *a priori* from drug molecule characteristics and from *in vitro* measures of metabolism, passive and active transport, and protein binding, we are at present hampered by the lack of recognition that intraorgan concentrations are ignored when we determine the apparent clearance from AUC and steady-state systemic concentrations.

There is only one valid mechanistic definition of clearance. Clearance is driven by exposure proximate to the elimination machinery and is always model dependent.

## Supplementary Information

ESM 1 Derivations of Eqs. 4-6 and 8 are provided. (DOCX 33.4 kb)

## Abbreviations

AUC: area under the curve
BCRP: breast cancer resistance protein
*C*_*in*_: concentration entering an organ of elimination
*C*_*out*_: concentration exiting an organ of elimination
*C*_*ss*_: steady-state concentration
*C*_*systemic*_: systemic concentration
*CL*: clearance
*CL*_*H*_: hepatic clearance
*CL*_*int*_: intrinsic clearance
CSTR: continuous stirred-tank reactor
DM: dispersion model
*ER*: extraction ratio
*F*: bioavailability
*f*_*u,B*_: fraction unbound to proteins in blood
HMG-CoA: 3-hydroxy-3-methyl-glutaryl-coenzyme A reductase
GFR: glomerular filtration rate
IPRL: isolated perfused rat liver
IVIVE: *in vitro*–*in vivo* extrapolation
*K*_*p,uu*_: ratio of unbound liver to blood concentration
NME: new molecular entity
PBPK: physiologically based pharmacokinetics
PFR: plug flow reactor
P-gp: P-glycoprotein
PTM: parallel tube model
*Q*_*H*_: hepatic blood flow
WSM: well-stirred model
