# Supplementary material for: There is Only One Valid Definition of Clearance: Critical Examination of Clearance Concepts Reveals the Potential for Errors in Clinical Drug Dosing Decisions
Source: AAPS J. 2021 May 10;23(3):67. doi: 10.1208/s12248-021-00591-z (PMC8110503; doi:10.1208/s12248-021-00591-z)
Supplement: Supplementary file 1 — Derivations of Eqs. 4-6 and 8 are provided. (DOCX 33.4 kb) [file 12248_2021_591_MOESM1_ESM.docx]

**Supplementary Material**

**There is Only One Valid Definition of Clearance:**

**Critical Examination of Clearance Concepts Reveals the Potential for Errors**

**in Clinical Drug Dosing Decisions**

**Derivation of Relevant Equations**

Here we show the validity of Eq. 4 in the manuscript for the two well-established reactor models, namely *WSM* and *PTM*, the first with infinite dispersion, the latter with zero dispersion. The dispersion number describes the spread in residence times of molecules within the reactor (or organ). From a chemical reaction engineering point of view, as shown in Fig. 1, *WSM* corresponds to a well-mixed *CSTR*, whereas *PTM* corresponds to a *PFR* where only convective flow is considered. The notation here follows the common chemical reaction engineering notation. Consider the steady-state equation for a *CSTR*

$QC_{in}-QC_{out} -k{VC}_{out}=0$ (S1)

which is a mass balance based on the assumption that the concentration is homogenous within the volume $V$ of the reactor, and $k$ is a first-order rate constant expressed in units of inverse time. The volumetric flow rate is $=UA$, where $U$ is the velocity at which liquid stream enters and exits the reactor (distance/time units) and $A$ is the cross-section of the inlet/outlet (units of area).

The total rate of reaction (r) in the *CSTR* (for the first-order kinetics considered) is defined as $\int_{V} rdV=kVC_{out}$. The integral over the volume is the general definition of the total rate of reaction, regardless of the model, although the definition of r will be model dependent. After simple algebraic manipulations, it can be shown that

$\frac{Total rate of Reaction}{Rate of Delivery to the Reactor}\left. \right|_{CSTR}=\frac{kVC_{out}}{UAC_{in}}=\frac{QC_{in}-QC_{out}}{UAC_{in}}\left. \right|_{CSTR}=\frac{{(C}_{in}-C_{out})}{C_{in}}\left. \right|_{CSTR}$ (S2)

Now, we derive the equivalent equation for the *PFR*. The mass balance for *PFR* is given by Fogler (8). Here, we present the solution of the equation and the keen reader is referred to the previous reference for a detailed derivation. The *PFR* has a length *L* and the mass balance is analytically solved, so that the concentration profile over the whole domain $z$ in $[0,L]$ is derived. Following a non-dimensionalization procedure, as commonly done in chemical engineering analysis, we can denote $\lambda=\frac{z}{L}$ as the dimensionless length. We also define the dimensionless concentration along the reactor length as $=\frac{c}{C_{in}}$. Let us define the Damkholer number ($Da$) as

$$\frac{k}{U/L}=Da$$

which compares the characteristic rate of reaction to the characteristic rate of convection. Accordingly, the concentration along the reactor varies as

$\Psi=exp(-Da\lambda)$

Therefore, by observing that $\Psi_{out}=\frac{c_{out}}{C_{in}}=exp(-Da)$, since the outlet concentration corresponds to $\lambda=1$, we arrive at

$\frac{k}{U/L}=\frac{{(C}_{in}-C_{out})}{{(C}_{in}-C_{out})/ln(\frac{C_{in}}{C_{out}})}$

The total rate of reaction in the *PFR* is given by

$$Total rate of Reaction=\int_{V} rdV=AL\int_{0}^{1} kC_{in}\exp\left( -Da \lambda^{'} \right)d\lambda^{'}=ALkC_{in}\frac{(1-\exp\left( -Da \right))}{Da}$$

$=(1-\exp\left( -Da \right))UAC_{in}$ (mol/s) (S3)

Moreover, the definition of the rate of delivery to the reactor does not change among *CSTR* and *PFR*, thus:

$\frac{Total rate of Reaction}{Rate of Delivery to the Reactor}\left. \right|_{PFR}=\frac{(1-\exp\left( -Da \right))UAC_{in}}{UAC_{in}}=(1-\exp\left( -Da \right))=\frac{{(C}_{in}-C_{out})}{C_{in}}\left. \right|_{PFR}$ (S4)

Therefore, it follows from above that Eq. 4 in the manuscript holds for the *CSTR* and *PFR* models (respectively, *WSM* and *PTM*).

*.*

As noted in the manuscript, each of the chemical reaction engineering models in Fig. 1 will exhibit different mean residence times for substrate within the reactor (8), although each of the models in Fig. 1 at steady-state have the same concentration in and the same concentration out values. Similarly, in pharmacokinetics, the different models will also exhibit different mean residence times for concentrations within the liver, although each of the models at steady-state have the same *C_in_* and the same *C_out_*. The mean residence time is defined as the average amount of time each molecule spends within a reactor or organ. Since the volume of distribution for drug inside the liver will be the same for each of the models, this difference in mean residence time will translate into different clearances, and *vice versa* for the models depicted in Fig. 1, as in pharmacokinetics, clearance is the ratio of volume of distribution to mean residence time (28). Rowland et al. (12) proposed that Eq. 5 defines the clearance of the well-stirred model (WSM) of Fig. 1A

${CL}_{H,WSM}=Q_{H}\cdot\frac{(C_{in}-C_{out})}{C_{in}}$ (5)

For the parallel tube and the dispersion models at steady-state the exposure driving elimination can be expressed in terms of the average concentration within the liver. This can be derived in pharmacokinetics for the parallel tube model as described below.

For the parallel tube model, elimination within the liver at steady-state follows first-order elimination, designated by the rate constant k. *C_out_* can be expressed in terms of the *C_in_* either as a function of distance within the liver (*L* from entry *0* to exit *L_out_*) between entering and exiting, but also in terms of time (*t* from entry *0* to exit *t_ou_*_t_) within the liver. It is well recognized in chemical reactor engineering that at steady-state, when the rate of flow is constant, distance and time within the reactor are not independent. This has only recently been pointed out in pharmacokinetics (29). The exposure within the liver for the parallel tube model at steady-state can be derived either in terms of distance or time, yielding the same result. Here, we derive the relationship in terms of time within the liver.

$C_{H}=C_{in}\cdot e^{-kt}$ (S5)

where *C_H_* is the concentration in the liver at any time t from entry. Then

$C_{out}=C_{in}\cdot e^{-kt_{out}}$ (S6)

Integrating between *0* and *t_out_* yields the exposure in terms of area under the curve within the liver (*AUC_H_*)

${AUC}_{H}=C_{in}\cdot\int_{0}^{t_{out}} e^{-kt}=C_{in}\cdot\frac{e^{-k t}}{-k}$ (S7)

which when evaluated at *0* and *t_out_* yields

${AUC}_{H}=\frac{C_{in}}{-k}\cdot(e^{-kt_{out}}-1)$ (S8)

But, from Eq. S6

$k=\frac{ln\frac{C_{out}}{C_{in}}}{t_{out}}$ (S9)

which when substituted into Eq. S8 yields

${AUC}_{H}=\frac{(C_{in}-C_{out})\cdot t_{out}}{ln\frac{C_{in}}{C_{out}}}$ (S10)

Clearance in the parallel tube model will equal the amount lost $Q_{H}\cdot(C_{in}-C_{out})\cdot t_{out}$ divided by the exposure. Therefore, dividing amount lost by Eq. S10 will yield the clearance for the parallel tube model (Fig. 1B) as given as Eq. 6 in the manuscript and recently presented (29)

${CL}_{H,PTM}=Q_{H}\cdot ln\frac{C_{in}}{C_{out}}$ (6)

Thus, it is obvious using a derivation for time within the organ of elimination, that the clearance for the parallel tube model will not be the same as for the well-stirred model. It is also obvious that $ln\frac{C_{in}}{C_{out}}$ does not equal the extraction ratio. The same result would occur using a derivation for distance within the organ of elimination, since C_in_ and C_out_ are neither a function of distance nor time in the clearance equations (12, 13).

In the early 1970s analyses were undertaken to characterize the effects of blood flow, protein binding and metabolic capacity to affect clearance leading to Eq. 8 for what was designated as the well-stirred model.

${CL}_{organ,WSM}=Q_{organ}\cdot\frac{f_{u,B}\cdot{CL}_{int}}{Q_{organ}+f_{u,B}\cdot{CL}_{int}}$ (8)

Although the original derivation was much more convoluted (12), it is quite easy to show the basis and assumptions inherent in Eq. 8. First assume that the model independent amount eliminated per unit time is equal to the product of the intrinsic clearance (*CL_int_*) of unbound drug within the liver multiplied by the unbound concentration in blood exiting the liver (*C_u,out_*).

$Q_{H}\cdot\left( C_{in}-C_{out} \right)={CL}_{int}\cdot C_{u,out}$ (S11)

The unbound concentration may be converted to total concentration in blood by inserting the fraction unbound parameter (*f_u,B_*).

$Q_{H}\cdot\left( C_{in}-C_{out} \right)={f_{u,B}\cdot CL}_{int}\cdot C_{out}$ (S12)

Now from the Rowland et al. (12) definition of the well-stirred model relationship of Eq. 5

$Q_{H}\cdot\left( C_{in}-C_{out} \right)={{CL}_{H,WSM}\cdot C_{in}=f_{u,B}\cdot CL}_{int}\cdot C_{out}$ (S13)

And then one can solve the two right hand terms of Eq. S13 for the ratio of exiting to entering concentrations.

$\frac{C_{out}}{C_{in}}=\frac{{CL}_{H,WSM}}{f_{u,B}\cdot{CL}_{int}}$ (S14)

One may also write the designated well-stirred model, Eq. 5, in terms of the ratio of exiting to entering concentrations

${CL}_{H,WSM}=Q_{H}\cdot\frac{\left( C_{in}-C_{out} \right)}{C_{in}}=Q_{H}-Q_{H}\cdot\frac{C_{out}}{C_{in}}$ (5)

Rearranged to give

$\frac{C_{out}}{C_{in}}=1-\frac{{CL}_{H,WSM}}{Q_{H}}$ (S15)

Then setting the right-hand side of Eq. S14 equal to the right-hand side of Eq. S15, and solving for *CL_H,WSM_* yields Eq. 8. Clearance and intrinsic clearance terms are not utilized in chemical reaction engineering, but one can recognize in Eq. S1 that the *kV* product in the CSTR model corresponds to the *CL_int_* term of the WSM. However, Eq. S3 defines the total rate of reaction for the PFR model and one can easily see that if an intrinsic clearance term were to be defined for this model, it must be related to *C_in_* and the Damkholer number, not to the simple *C_out_* term of the CSTR model. The error in the literature in attempting to define an intrinsic clearance for the parallel tube and dispersion pharmacokinetic models is the belief that the intrinsic clearance relationship based on the WSM model will predict the clearance for these alternate models. The field of pharmacokinetics has not realized that the definition and relevance of intrinsic clearance only relates to the well-stirred model and that the relationship between liver clearance and intrinsic clearance for any alternative model of organ elimination has not been derived, although many papers assume to have done so, because they assume that Eq. 5 is model independent. Equation S11 provides a useful approach for analyzing the well-stirred model because in the well-stirred model and only for the well-stirred model

${CL}_{H,WSM}\cdot C_{in}={CL}_{int}\cdot C_{u,out}$ (S11A)

Therefore, in the well-stirred model it is possible to use this relationship where elimination is assumed to be driven by the exiting unbound concentration, because in fact this product is equivalent to the product on the left-hand side of Eq. S11A. That is, although Eq. 5 is not consistent with the pharmacokinetic definition of clearance (amount eliminated divided by the exposure driving that elimination), the invention of intrinsic clearance by Rowland et al. (12) and the equivalence shown in Eq. S11A, allows Eq. 5 to be consistent with the well-stirred model in pharmacokinetics and the CSTR model of chemical reaction engineering. However, as can be seen for the derivations above, this equivalence will not hold for any other model of hepatic elimination. That is, if it was possible to derive *CL_int_* for alternate models of hepatic elimination, the product ${CL}_{int}\cdot C_{u,out}$ of would not be equal to clearance via that model multiplied by *C_in_*, contrary to the assumption of the model independence of Eq. 3, the 1972 relationship of Rowland (1).

**References**

28. Benet LZ, Galeazzi R. Noncompartmental determination of the steady-state volume of distribution. J Pharm Sci. 1979;68:1071–4.

29. Kochak G. Critical analysis of hepatic clearance based on an advection mass transfer model and mass balance. J Pharm Sci. 2020;109:2059–69.
